# The Effect of Type-2 Diabetes on Cognitive Status and the Role of Anti-diabetes Medications

**DOI:** 10.7759/cureus.19176

**Published:** 2021-11-01

**Authors:** Almothana Alkasabera, Chike B Onyali, Comfort Anim-Koranteng, Hira E Shah, Aarthi Ethirajulu, Nitin Bhawnani, Jihan A Mostafa

**Affiliations:** 1 General Medicine, California Institute of Behavioral Neurosciences & Psychology, Fairfield, USA; 2 Internal Medicine, EHA Clinics, Abuja, NGA; 3 Medicine, California Institute of Behavioral Neurosciences & Psychology, Fairfield, USA; 4 Internal Medicine, California Institute of Behavioral Neurosciences & Psychology, Fairfield, USA

**Keywords:** alzheimer disease, anti-diabetics, cognitive decline, insulin, amyloid plaques, type 02 diabetes mellitus, insulin resistance

## Abstract

Type-2 diabetes mellitus prevalence is constantly increasing; this is explained by the increase of its risk factors and the amelioration of its management. Therefore, people are living longer with diabetes mellitus, which, in turn, has revealed new complications of the disease. Dementia is represented mainly by Alzheimer's disease and is an interesting topic of study. Accordingly, statistics have shown that dementia incidence is doubled in diabetic patients.

The establishment of a relation between type-2 diabetes mellitus was studied on several levels in both humans and animal subjects. First, insulin receptors were found in the brain, especially the hippocampus, and insulin transport to the brain is mainly accomplished through the blood-brain barrier. Secondly, several studies showed that insulin affects multiple neurotransmitters in favor of promoting memory and cognition status. Thirdly, multiple pathological studies showed that insulin and Alzheimer's disease share many common lesions in the brain, such as beta-amyloid plaques, amylin-Aβ plaques, hyper-phosphorylated tau protein, and brain atrophy, especially in the hippocampus. After recognizing the positive effect of insulin on cognitive status, and the harmful effect of insulin resistance on cognitive status, multiple studies were focused on the role of anti-diabetes medications in fighting dementia. Consequently, these studies showed a positive impact of oral anti-diabetes medication, as well as insulin in limiting the progression of dementia and promoting cognitive status. Moreover, their effects were also noticed on limiting the pathological lesions of Alzheimer's disease.

Accordingly, we can consider type-2 diabetes mellitus as a risk factor for dementia and Alzheimer's disease. Therefore, this can be used on the pharmaceutical level by the promising implication of antidiabetics as a treatment of dementia and Alzheimer's disease or at least to limit its progression. However, multiple clinical studies should be dedicated to proving the true benefits of anti-diabetes medications in treating dementia before they can be used in reality.

## Introduction and background

Type-2 diabetes mellitus (T2DM) is featured by chronic resistance to insulin and high blood glucose. Furthermore, Its long-term complications affect the kidneys, the retina, the peripheral neurons, the heart, and blood vessels [1,2]. High body mass index along with increased population median age is making type-2 diabetes mellitus more prevalent [3]. T2DM undergoes the fastest increase in prevalence among all other diseases, with 410 million patients in 2013, which represents a 133% increase in the last 13 years before that date [4]. Moreover, this incidence will continue rising as risk factors for this disease also proceed to rise in the community, including overnutrition and malnutrition, sedentary lifestyle, and low physical activity, stress, and many other socioeconomic and environmental factors [5,6]. In addition, the amelioration of the management protocols of diabetes and diabetic-related micro- and macro-vascular complications, as well as preventive efforts, had led to the fact that patients are now living longer with diabetes mellitus, which might lead not only to increased prevalence but also lead to the unveiling of new complications. One example of these complications is dementia, especially in the form of Alzheimer's disease. Accordingly, age may play a role in the occurrence of the two diseases simultaneously. According to the 2017 estimate of the prevalence of diabetes in people aged 65 or above was 18.8, which equals 122.8 million [7] in the whole world. As we mentioned earlier, this is subjected to increase in the subsequent decades as a result of increasing age [7]. In correlation, dementia numbers submit to the same equation with a prevalence of 6%-7% in people aged 60 or above [8], along with 46.8 million people living with dementia around the world, with an expected doubling of this number in the next two decades according to the 2015 estimate [8]. Several prospective studies revealed that in type-2 diabetes, the risk of the co-occurrence of dementia is doubled [9-13], including Alzheimer's disease, which is by far the most common cause of dementia [14].

Alzheimer’s disease (AD) is characterized by the accumulation of beta-amyloid plaques along with hyper-phosphorylated tau protein, which will lead to the progressive degeneration of the neurons in the brain. This is translated clinically by progressive dementia and cognitive decline, with death as a certain end of this disease [15]. Dementia is most commonly caused by Alzheimer's disease, which accounts for 60%-80% of all dementia cases [16]. AD cases are expected to reach a prevalence of 80 million by 2040 in the whole world [17]. Regarding the pathology of AD, we have two main aspects. On the one hand, we have the amyloid-beta (Aβ) plaques that come from the amyloid precursor protein (APP), which will be overproduced. On the other hand, we have the neurofibrillary tangles, which are formed by phosphorylated tau protein that comes from paired helical filaments (PHFs) [18-20].

The relationship between diabetes and dementia is still obscure, and many efforts have been gathered lately to illustrate the link between diabetes and cognitive decline and to what extent can antidiabetics help in treating dementia, prevent it or even hold its progression. In this review, we will see the relationship between type-2 diabetes mellitus and dementia on multiple levels. In addition, we will also review the effects of anti-diabetes medication upon this relation.

## Review

Cognition and insulin receptors distribution in the brain

Insulin receptors are diffusely spread among all cells of the brain. However, there are some variations in its expression in the different sits of the brain. Moreover, it's found that insulin receptors concentrate the most in the olfactory bulb, hypothalamus, hippocampus, cerebral cortex, striatum, and cerebellum [21-24]. Therefore, the existence of insulin receptors in this way must serve the function of these areas.

If we look in the neuron itself, we find that insulin receptors are concentrated in the neuronal soma and even more in the synaptic terminals, which surely, by example if we consider the hippocampus, aid in the function of maintaining memory in it [24-26].

In the review of the above, we know that insulin receptors do exist in the brain in a matter that is functionally related to cognitive function in a strong way.

Insulin levels in the brain and what all does it affect?

A lot of effort has been made to determine where does the insulin in the brain comes from. On the one hand, it is suggested that it may come from outside the brain through the blood-brain barrier across certain saturable transporters [27,28]. However, this transport is found to be influenced by obesity, serum triglycerides level, inflammation, glycaemia status, and diabetes mellitus [28].

On the other hand, it may come from the brain itself, which in this case follows an autocrine mode through insulin production by the olfactory bulb and the dentate gyrus, to serve its effects on the olfactory bulb and the hippocampus [29-31]. This was evidenced by finding c peptide, which is an insulin by-product substance, in multiple human brain regions [32,33]. In contrast, multiple human studies have failed to report insulin mRNA in the brain. Nevertheless, it is worth mentioning that the amount of insulin in the CSF is much less than the amount of insulin in the serum; although their rates are correlated, thus we know that the most important source of the brain's insulin is its transport through the blood-brain barrier [34,35]. 

Interestingly, the amount of insulin was found to be decreased in the post-partum brains of Alzheimer's disease patients [36]. Additionally, compared to the CSF of normal subjects, a decreased CSF insulin in type-2 diabetes mellitus patients (insulin resistance state) was noticed, which can be explained by decreased transport across the blood-brain barrier [37].

In the review of the above, we understand that insulin does not only come from serum insulin (pancreatic insulin) but also is expressed and produced by neurons of the brain to a lesser extent. It's also noticed that in both diseases: Alzheimer's disease and diabetes mellitus, there is evidence of decreased insulin levels in the brain compared to normal people.

How does type-2 diabetes mellitus functionally relate to dementia?

Insulin signaling cascade in improving synaptic plasticity in the hippocampus, which is primordial in memory functioning, is now becoming more and more familiar.

Mammalian brain insulin was proven to help in hippocampal long-term potentiation (LTP), which is essential in memory function [38]. In addition, insulin was also found to exert certain effects on multiple neurotransmitters that are implicated in memory formation, like acetylcholine, norepinephrine, and epinephrine [39]. Furthermore, insulin was also found to increase (gamma-aminobutyric acid) GABA and N-methyl-D-aspartate receptors [38,40].

Further relations between dementia and type-2 diabetes mellitus have been established using brain magnetic resonance apectroscopy on diabetic patients, which has revealed multiple metabolic abnormalities criteria of dementia in diabetic patients' brains. For instance, human-based studies regarding the cognitive decline in type-2 diabetes mellitus patients revealed the following: extremely decreased N-acetylaspartate (NAA) levels, which contribute to altered neuronal integrity, increased myoinositol levels, elevated levels of excitatory neurotransmitters, including glutamate and glycine, and decreased levels of inhibitory neurotransmitters, including GABA which is also implicated with pain's perception issues [41].

Additionally, after inducing diabetes in rats and examining their brains, we notice myelin degeneration and multiple vacuoles distributed in the white matter along with brain atrophy [42]. Furthermore, more than 50% reduction in the levels of tyrosine and phenylalanine, which serve as a precursor of catecholamines, is noticed too [43].

In the review of the above, we conclude that diabetic patients suffer from neurotransmitters' metabolic abnormalities in the brain, which eventually will lead to neuronal malfunctioning and destruction, which in turn will eventually progress to dementia.

How does type-2 diabetes mellitus pathologically relate to dementia?

The possibility of establishing a connection between type-2 diabetes mellitus and Alzheimer's disease through some pathological changes that overlap between the two diseases was always attended.

We found that type-2 diabetes mellitus patients have amylin aggregations in the pancreatic islets, kidneys, and the heart [44-46]. Furthermore, the brain of type-2 diabetes mellitus patients along with Alzheimer's disease patients was found to have an appreciable amount of amylin aggregations along with what is called mixed amylin-Aβ plaques in their brains [47-51].

Similarly, animal-based trials have shown that insulin resistance is linked to increased production of Aβ plaques and hyper-phosphorylation of tau protein [52-61]. Insulin deficiency and diabetes induction in mice through the use of streptozotocin has been beneficial to show increased brain levels of hyper-phosphorylated tau protein in these animals [52,53,55,62]. Insulin resistance was found to have a contribution in promoting Aβ plaques, which was explained by malfunctioning of Aβ removing from the brain, due to increased beta-site amyloid precursor protein cleaving enzyme (BACE)1 over β-secretase and γ-secretase activities [58,59].

Moreover, diabetic-induced rats have developed demyelination along with multiple vacuoles in the white matter and overall brain atrophy [42]. Another study used MRI to assess the brains of rats and humans that suffered from impaired glucose regulation revealed a hypotrophic hippocampus in both animals models [63] and human subjects [64-68].

In the review of the above, diabetic patients may have some of the principal pathological features of Alzheimer's disease developed over the years, and they may also have some of the diabetes type amyloidosis aggregations in the brain.

**Figure 1:**
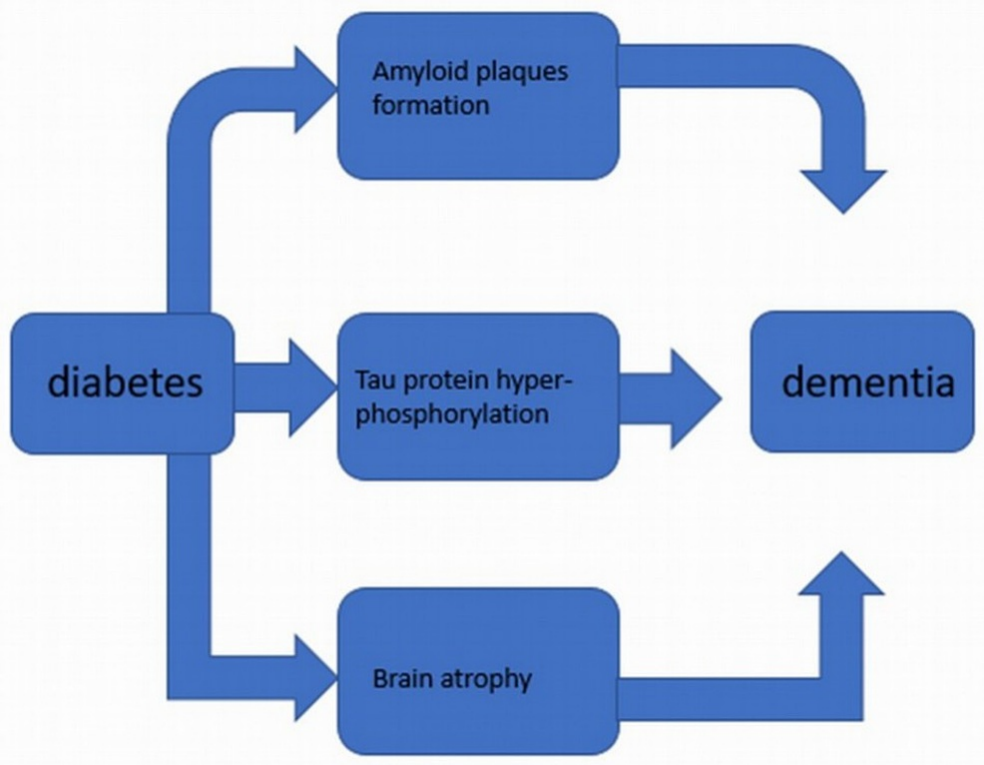
pathological changes in diabetics, which lead to dementia

The studies that are used to gather this information are animal (mostly rats) and human-based. However, human-based studies are more accurate and express little bias compared to animal-based studies. Figure 1 illustrates the major pathological consequences of diabetes that contribute to dementia. 

Diabetic medications' influence on cognitive status

Metformin works by reducing hepatic gluconeogenesis and enhancing insulin sensitivity in tissues. Metformin crosses the blood-brain barrier fast [69], and its overall effect is suggestive of being neuro-protective and anti-neuronal aging by promoting mitochondrial function [70,71]. Human-based observational studies have revealed that diabetic patients who took metformin have revealed improvement of dementia symptoms [72,73] and minimizing of mild cognitive impairment [74] when compared to patients who don’t take any medication or taking other antidiabetics. 

Sulfonylureas increase insulin secretion by blocking potassium channels on beta cells. However, glimepiride was found to decrease the effect of amyloid on synaptic degeneration in vitro [75]. Furthermore, gliclazide acted as an antioxidant in the brain of diabetic rats [76]. 

Thiazolidinediones (Glitazones) increase insulin sensitivity by activating peroxisome proliferator-activated receptor-γ in the nucleus. It is important to note that pioglitazone is the only thiazolidinedione available in the market that crosses the blood-brain barrier. Pioglitazone effects in mice after four months of treatment are as follows: decreased tau hyper-phosphorylation, decreased amyloid levels, memory deficits, and finally decreased spatial learning limitations [77].

Glucagon-Like Peptide-1 (GLP-1) Receptor Agonists are incretins secreted by the intestines, and they slow gastric motility and increase insulin secretion and decrease glucagon secretions. Glucagon-Like Peptide-1 (GLP-1) Receptor Agonists cross the blood-brain barrier, and its receptors have been found in the brain, especially the hippocampus [78]. Alzheimer's disease mice models treated with GLP-1 exerted a neuro-protective effect against apoptosis, oxidative stress, and decreased synaptic plasticity in the hippocampus caused by beta-amyloid [79,80].

Dipeptidyl Peptidase-4 Inhibitors (DPP-4i) work by inhibiting the DPP-4 enzyme that proteolysis GLP-1. In animal-based studies, Alzheimer's disease-induced animals' given DPP-4 inhibitors have shown decreased tau protein phosphorylation along with decreased amyloid plaques [81,82]. Human-based studies in the elderly, who are type-2 diabetics and have mild cognitive decline, and treated with DPP-4 inhibitors, have revealed no further decline in their cognitive status [83].

Insulin, when used parenterally, does concentrate in the cerebrospinal fluid in appreciable amounts because of the saturable transfer across the blood-brain barrier, and the risk of hypoglycemia becomes a limitation when attempting to increase the dose. However, when given intranasally, it bypasses the blood-brain barrier, and we can reach an appreciable amount of it in the brain [84]. Insulin in vivo studies has shown that it helps in the regulation of tau protein phosphorylation, the metabolism of the beta-amyloid precursor protein, and the removal of beta-amyloid [85]. Moreover, intranasal insulin promotes memory function in healthy, mild cognitive impairment, and advanced Alzheimer's disease patients. It also preserves the volume of the brain regions affected by Alzheimer's disease [86]. Table 1 is a comparison between different medications of diabetes according to its antidementia and antidiabetic effects.

**Table 1:** Various anti-diabetes medications' effect upon diabetes and dementia DDP4-i: Dipeptidyl Peptidase-4 Inhibitors, GLP-1: Glucagon-Like Peptide-1

Medication	Anti-diabetic effect	Anti-dementia effect
Metformin	Decreases gluconeogenesis and increases insulin sensitivity	Increases mitochondrial function
Sulfonylureas	Increase insulin secretion	Decrease oxidative stress
Glitazones	Increase insulin sensitivity	Decrease tau protein phosphorylation and decrease amyloid
GLP-1	Increases insulin secretion	Decreases apoptosis, decreases oxidative stress, and increases synaptic plasticity
DPP-4i	Increase insulin secretion	Decrease tau protein phosphorylation and decrease amyloid
Insulin		Decreases amyloid and decreases brain atrophy

Limitations

The limitations to this review are the lack of long-term and diverse clinical human-based trials of antidiabetic medications on Alzheimer's disease and other dementia patients, and the lack of evidence on knowing the exact benefits of the implication of anti-diabetes medications in treating dementia, especially Alzheimer's disease on the long run.

## Conclusions

The effect of type-2 diabetes on cognitive status can be answered by first proving that insulin and insulin receptors do exist in the brain and follow a certain pattern to serve a certain function in promoting cognitive status. However, type-2 diabetes mellitus effects were found to be in favor of dementia, and cognitive decline, on the metabolic level and the pathologic level. As this connection has been attempted to be made, we have found that anti-diabetic medication has also an effect upon cognitive status in a positive way, according to multiple animals and human-based studies. Nevertheless, we need more clinical trials before judging the true affectivity of these medications against dementia, especially Alzheimer's disease.
